# Effects of the prospective payment system on anemia management in maintenance dialysis patients: implications for cost and site of care

**DOI:** 10.1186/s12882-016-0267-x

**Published:** 2016-05-26

**Authors:** James B. Wetmore, Spiros Tzivelekis, Allan J. Collins, Craig A. Solid

**Affiliations:** Chronic Disease Research Group, Minneapolis Medical Research Foundation, 914 South 8th Street, Suite S4.100, Minneapolis, MN 55404 USA; Global Health Economics, Amgen, Inc., Thousand Oaks, CA USA; Department of Medicine, University of Minnesota, Minneapolis, MN USA

**Keywords:** Anemia, Dialysis, End-stage renal disease, Health economics, Medicare, Red blood cell transfusions

## Abstract

**Background:**

The 2011 expanded Prospective Payment System (PPS) and contemporaneous Food and Drug Administration label revision for erythropoiesis-stimulating agents (ESAs) were associated with changes in ESA use and mean hemoglobin levels among patients receiving maintenance dialysis. We aimed to investigate whether these changes coincided with increased red blood cell transfusions or changes to Medicare-incurred costs or sites of anemia management care in the period immediately before and after the introduction of the PPS, 2009–2011.

**Methods:**

From US Medicare end-stage renal disease (ESRD) data (Parts A and B claims), maintenance hemodialysis patients from facilities that initially enrolled 100 % into the ESRD PPS were identified. Dialysis and anemia-related costs per-patient-per-month (PPPM) were calculated at the facility level, and transfusion rates were calculated overall and by site of care (outpatient, inpatient, emergency department, observation stay).

**Results:**

More than 4100 facilities were included. Transfusions in both the inpatient and outpatient environments increased. In the inpatient environment, PPPM use increased by 11–17 % per facility in each quarter of 2011 compared with 2009; in the outpatient environment, PPPM use increased overall by 5.0 %. Site of care for transfusions appeared to have shifted. Transfusions occurring in emergency departments or during observation stays increased 13.9 % and 26.4 %, respectively, over 2 years.

**Conclusions:**

Inpatient- and emergency-department-administered transfusions increased, providing some evidence for a partial shift in the cost and site of care for anemia management from dialysis facilities to hospitals. Further exploration into the economic implications of this increase is necessary.

## Background

Controlling health care costs while providing quality care for individual patients and for the population as a whole is a goal of payers such as the Centers for Medicare & Medicaid Services (CMS). The United States employs a variety of billing systems for billing for health care services; two of the main categories are fee-for-service billings, in which costs for services are billed separately as line items in environments such as the emergency department (ED), and bundled payment billings, in which a diagnosis-related group (DRG) is billed as a set amount for a particular type of hospitalization (such as an admission for congestive heart failure). Costs related to end-stage renal disease (ESRD) patients receiving maintenance dialysis have been afforded special scrutiny by CMS, since these costs are vastly disproportionate to ESRD patients’ representation in the Medicare population. In 2011, CMS introduced the Prospective Payment System (PPS, or “the dialysis bundle”), an expanded capitated payment system encompassing a range of dialysis-related products and services [1]. This PPS was designed to create incentives for dialysis providers to control costs, especially for medications such as erythropoiesis-stimulating agents (ESAs) used to treat ESRD-related anemia. In so doing, CMS sought to decrease overall expenditures associated with the ESRD program by approximately 2 %. To help ensure that the PPS did not negatively affect patient care outcomes, CMS contemporaneously established the Quality Improvement Program [2]; this was initially designed to protect against the possibility that hemoglobin levels would drop unduly, although the metric for hemoglobin below 10 g/dL was later eliminated following a 2011 Food and Drug Administration ESA label revision [3].

While the temporal relationship between the introduction of the PPS and patterns of anemia management has been partially explored [4–7], the extent to which less use of ESAs may be associated with more use of red blood cell (RBC) transfusions or an associated increase in transfusion-related costs is not fully established. Additionally, because the PPS does not include costs incurred outside the outpatient dialysis setting (e.g., those associated with hospitalization or outpatient transfusion centers), it is unclear whether costs and sites of care of anemia management may have partly shifted from one setting to other, more intensive settings (e.g., the ED).

Accordingly, we sought to examine how use of ESAs, intravenous (IV) iron, and RBC transfusions, along with their associated costs to Medicare, changed in the period immediately before and after introduction of the PPS. To do so, we used data from 2009 to 2011. At the same time, we sought to determine whether RBC transfusion rates were increasing in inpatient, outpatient, or ED settings. We hypothesized that while use of ESAs likely declined, as shown by others, use of IV iron and RBC transfusions, along with their associated costs, likely increased; if true, this would likely have the effect of not only increasing the use of blood products, a limited societal resource, but also of shifting some of the anemia management burden from dialysis providers to the institutions that administer most RBC transfusions, such as hospitals [4]. We reasoned that understanding the effects of the PPS could be important when designing future reimbursement policies or payment models, such as ESRD-specific accountable care organizations, aimed at controlling costs.

## Methods

### Subjects and data source

This study used US Medicare ESRD registry files with Part A and Part B Medicare claims for ESRD patients on maintenance hemodialysis. As the goal was to study the potential effects of the PPS, we used data in the period immediately before and after its introduction, namely 2009–2011 (the last year for which data were available for analysis). The CMS Medicare ESRD standard analytic files (SAFs) were used to obtain information on Medicare patients. Demographic information was obtained from the Medical Evidence Report (form CMS-2728). Facility information was obtained from dialysis claims data. This was a descriptive study to ascertain whether any important associations could be detected; no statistical modeling was undertaken.

The study population consisted of facilities that enrolled 100 % into the ESRD PPS in 2011 and had Medicare primary insured ESRD patients with Part A and Part B coverage on maintenance dialysis, and patients who dialyzed at those facilities. Of note, regulations permitted facilities to transition gradually (25 % per year over 4 years) to the PPS. The 203 potentially eligible facilities (4.2 % of the total) that chose this option were excluded from the analysis because we reasoned that requiring uniformity in the PPS adoption strategy would yield the most informative analysis. Facilities were also excluded if an ownership change occurred during the study period (2009–2011). Patients were attributed to only one facility at which they dialyzed most frequently during each quarter. Patient characteristics across all facilities included age, sex, race, and dialysis duration. Characteristics were calculated at the facility level, with medians and quartiles of the measured distributions reported. For example, for age, the median reported represents the value at which the median age was older at half of the facilities and younger at half.

### Outpatient transfusions and related costs

Using previously published [5, 8] methods, we searched Medicare claims for outpatient transfusions and related services, including transfusion-related adverse events. No attempt was made to determine the specific indication for transfusion. These services included pre- and post-transfusion screening (antibody, chemistry, hematology, immunology) within 3 days before and after the transfusion, and acquisition and administration of blood products and storage. We also identified transfusion-related adverse events, including febrile non-hemolytic reaction, hemolytic reaction, air embolism, phlebitis, hyperkalemia, allergic reaction, congestive heart failure (CHF), transfusion-related lung injury, and transfusion related circulatory overload [8], within 3 days of the transfusion and delayed hemolytic reaction within 45 days. CHF hospitalizations were included as adverse events only for patients with no other CHF-related episodes in the 6 months before the transfusion. Transfusion-related costs represented Medicare-reimbursed amounts on claims for transfusions and transfusion-related services, and were attributed to a facility through the patients attributed to that facility. Outpatient dialysis-related Medicare-reimbursed amounts included only those accrued at the facility to which patients were attributed (i.e., infrequent dialysis treatments at a different facility were not included as costs to the patient’s attributed facility). However, Medicare-reimbursed amounts for all outpatient transfusions and complications requiring hospitalization that occurred at other facilities were attributed to the patient’s attributed facility. Outpatient dialysis-related Medicare-reimbursed amounts included composite rate and the injectable medications ESAs, IV iron, IV vitamin D, and IV antibiotics. Medicare-reimbursed amounts were summed quarterly on a facility level, as was total patient time at risk for accruing costs, and a per-patient-per-month (PPPM) cost to Medicare was calculated for each facility for each quarter. Quarterly amounts were adjusted to December 2011 dollars using the Medical Consumer Price Index [9].

### Anemia management

Information on ESAs and IV iron doses was obtained from Medicare claims. Hemoglobin concentrations were obtained from monthly outpatient dialysis claims, and during each month and quarter we calculated the percentage of patients at each facility with at least one reported hemoglobin concentration below 10 g/dL.

Anemia management measures were calculated on a facility level, so the distribution of facility-level summary measures is presented. For example, for mean IV iron dose, the median represents the value at which the mean dose was higher at half of the facilities and lower at half. Similarly, for the percentage of patients whose hemoglobin concentrations were below 10 g/dL, the median represents the value at which the percentage was higher at half of the facilities.

### Site of service

To ascertain evidence of a shift in site of care for RBC transfusions, claims for outpatient transfusions were categorized as from three sites of service: the ED, observation stays, and all other outpatient sites. ED visits were identified using revenue center codes 0450-0459 and 0981 on outpatient claims. Observation stays were identified using Healthcare Common Procedure Coding System codes G0378 and G0379 and revenue center codes 0760 and 0762. Unlike the previous measures, transfusion rates were calculated across all patients included in the analysis, not at the level of the facility; this was done to more clearly investigate rates as they related to site of service, regardless of the practice patterns of the dialysis facilities at which patients were dialyzing. The overall PPPM number of transfusions by site (including inpatient transfusions) represents the total number of transfusion claims during the quarter across all patients divided by the total patient time at risk during the quarter. This approach allows for more direct comparison of the sites of transfusions not occurring at the outpatient dialysis facility.

We applied to and received approval from the Human Subjects Research Committee of the Hennepin County Medical Center/Hennepin Healthcare System, Inc.

## Results

### Facility characteristics

For each quarter of 2009 through 2011, between 4141 and 4425 dialysis facilities (representing 70.6 %-75.4 % of all dialysis facilities) met eligibility criteria with at least 10 eligible patients. Characteristics of patients at these facilities are displayed in Table 1. In general, characteristics of facilities and the patients they served remained relatively constant over the study period. Facilities were similar regarding age and sex. The race distribution, however, varied substantially across facilities; the twenty-fifth and seventy-fifth percentiles of white patients ranged from 32.6 % to 86.6 %.

**Table 1:** Distribution of characteristics among the US dialysis facilities that opted in to the revised prospective payment system, first quarter of 2009 and last quarter of 2011

	Quarter and Year	
Characteristics	Q1 2009	Q4 2011
Number of facilities	4141	4363
Number eligible patients per facility		
Mean across facilities	49.12	47.05
25th percentile	28	27
Median	44	42
75th percentile	63	61
Median age of eligible patients per facility, years		
25th percentile	60.0	60.5
Median	64.0	64.0
75th percentile	67.5	68.0
Percentage of female patient per facility		
25th percentile	40.0	40.0
Median	45.7	45.7
75th percentile	51.4	51.5
Percentage of white patient per facility		
25th percentile	33.3	32.6
Median	63.6	62.5
75th percentile	86.6	85.7
Percentage of facilities in each region		
Northeast	13.1	12.5
South	46.3	46.1
Midwest	23.2	23.1
West	17.5	18.2

*Q* calendar quarter

### Overall medicare costs

**Fig. 1:**
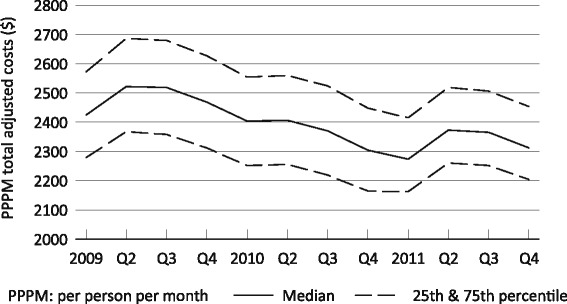
Per person per month adjusted total costs for outpatient dialysis and related injectable medications in US dialysis facilities that opted in to the revised prospective payment system, 2009–2011

The distribution of total PPPM Medicare reimbursement for dialysis-related services is illustrated in Fig. 1. With the introduction of the PPS, reimbursement amounts for injectable medications decreased, by definition, to zero. Median payment to facilities decreased slightly when adjusted for inflation (even with a simultaneous increase in the PPS composite rate), and Medicare-reimbursed amounts for outpatient transfusions and related activities were also relatively stable over the study period, increasingly slightly from $5.20 ± $11.51 per patient per month in Q1 2009 to $5.68 ± 13.71 in Q4 2011. Taking Q1 2011 as an example, we found the mean amount reimbursed for acquisition and administration to be $391, for screening and monitoring $113, and for the adverse events specified above $20, for a total of $524, a sum that changed little regardless of the quarter analyzed. While this is somewhat lower than previously reported [8], those investigators used data from employer group health plans, which are likely to reimburse at higher rates than Medicare.

### Anemia management

**Fig. 2:**
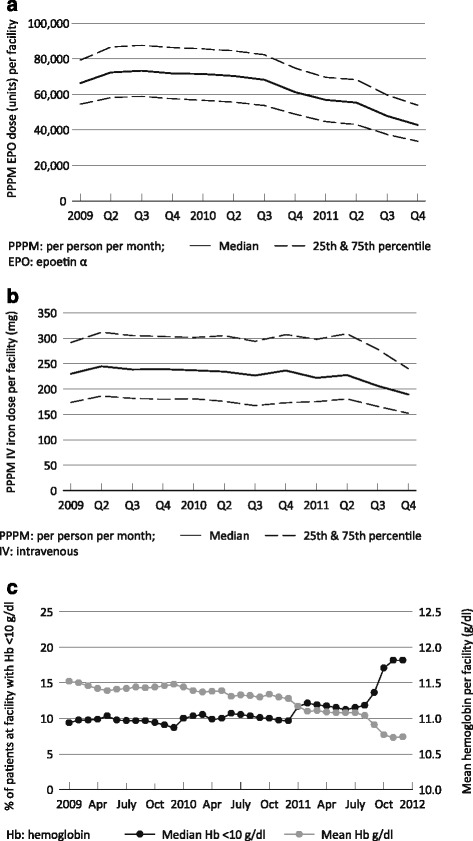
Distribution of facility-level anemia management parameters among patients dialyzing in US dialysis facilities that opted in to the revised prospective payment system, 2009–2011. Panel **a**, epoetin alfa per person per month dose per facility; panel **b**, intravenous iron per person per month dose per facility; panel **c**, hemoglobin concentrations per facility (black line, percentage of patients with hemoglobin concentrations < 10 g/dL at the facility; grey line, mean hemoglobin concentration, in g/dL, per facility)

The distribution of mean epoetin alfa (EPO) dose PPPM administered per facility declined steadily from the second quarter of 2010 (median 70,317 units) to the end of 2011 (median 42,769 units; Fig. 2a). The pattern of median PPPM number of IV iron administrations per facility increased from 2.37 in 2010 Q4 to 2.66 and 3.06 during the initial two quarters of the PPS (2011 Q1 and Q2), but then decreased in the last two quarters of 2011 to 2.92 and 2.63, respectively; concurrently, the distribution of mean dose PPPM administered per facility declined during the entire study period (Fig. 2b). Among patients receiving IV iron, the median dose per quarter dropped from 875 mg in Q4 2010 to 650 mg in Q4 2011 (data not shown). The distribution of mean monthly hemoglobin concentrations declined over time from 11.54 g/dL (twenty-fifth, seventy-fifth percentiles, 11.3, 11.8) in January 2009 to 10.76 g/dL (10.4, 11.1) in December 2011. The distribution of the percentage of patients with hemoglobin concentrations below 10 g/dL at each facility increased from a median of 9.4 % to 18.2 % (Fig. 2c).

### Frequency of transfusions and site of service

**Fig. 3:**
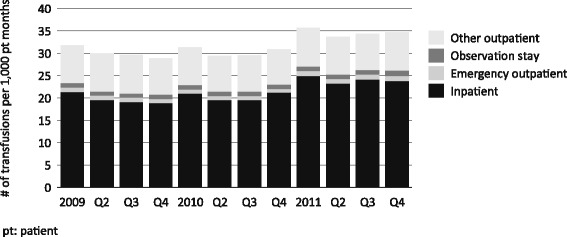
Red blood cell transfusion rates, by site of service among patients dialyzing in US dialysis facilities that opted in to the revised prospective payment system, 2009–2011

The number of inpatient transfusions appeared to be higher in 2011 than in 2009 or 2010 (Fig. 3), as did the number of outpatient transfusions (Table 2). Compared with the first quarter of 2009, the median of the distribution of the PPPM number of inpatient transfusions per facility was between 11 % and 17 % higher in each quarter of 2011, and the median percentage of patients per facility with at least one inpatient transfusion was between 7 % and 13 % higher in 2011 than in Q1 of 2009. (Of note, because reimbursement to inpatient facilities is based on the DRG system, we could not measure costs associated with transfusions occurring in the inpatient setting.)

**Table 2:** Number of transfusions per 1000 patient-months among patients dialyzing in US dialysis facilities that opted in to the revised prospective payment system, 2009–2011, by site of transfusion service

	Quarter and Year					
Service Site	2009 Q1	2009 Q4	2010 Q1	2010 Q4	2011 Q1	2011 Q4
Emergency department	0.99 (-)	0.92 (-7.4)	0.98 (-1.0)	0.88 (-10.3)	1.03 (3.7)	1.13 (13.9)
Observation stay	0.94 (-)	0.90 (-3.9)	0.93 (-0.9)	0.98 (4.2)	1.07 (14.4)	1.19 (26.4)
Other outpatient	8.46 (-)	8.11 (-4.1)	8.41 (-0.6)	7.82 (-7.5)	8.59 (1.5)	8.59 (1.6)
Inpatient	21.40 (-)	18.91 (-11.7)	21.00 (-1.9)	21.18 (-1.0)	24.96 (16.6)	23.81 (11.3)

Cell values represent transfusion rate (percentage change from 2009 Q1)

*Q* calendar quarter

An additional analysis was conducted to more precisely determine the site of outpatient transfusions (e.g., in the ED, during an observation stay, or in another outpatient setting); this is shown in Table 2. The number of transfusions in the ED increased by 0.14 per 1000 patient-months, in the observation stay environment by 0.25 per 1000 patient months, in other outpatient environments by 0.13 per 100 patient-months, and in the inpatient setting by 2.41 per 1000 patient-months. Although the number of overall outpatient transfusions PPPM increased slightly (5.0 %) from Q1 2009 to Q4 2011, the increase was 13.9 % in the ED and 26.4 % during an observation stay. Because observation stay and ED transfusions represent only 19.2 % of all outpatient transfusions during the study period, these increases did not translate into significant outpatient transfusion cost increases.

## Discussion

In this study, we sought to examine how the introduction of the expanded PPS and the ESA label change were associated with temporal changes in the patterns of ESA, IV iron, and RBC transfusion use, and to examine the costs and sites of care associated with anemia management in the period immediately before and after adoption of the PPS and the label change. This is an important issue; costs saved under the capitated PPS system, such as those for transfusions, could be shifted to hospitals because under the DRG system a hospital receives no additional payment, beyond that specified by the DRG, for a blood transfusion.

Overall Medicare payments to outpatient dialysis facilities for dialysis-related services declined on a PPPM basis from 2009 (pre-bundle) through 2011 (post-bundle), a finding consistent with previous work [10]. However, we also found increasing use of transfusions in the inpatient and, especially, observation stay and ED settings. Overall, these changes were modest, increasing by 0.25 per 1000 patient-month or less in the ED or during observation stays. Nevertheless, this phenomenon may represent a shift in the costs and sites of care for anemia management from the dialysis unit to more the expensive hospital-based care environment.

Collectively, it appears that the introduction of the PPS and the ESA label change have been associated with changes in anemia management in important ways. Patients dialyzing at the facilities we studied had lower hemoglobin concentrations on average, and more patients had concentrations below the threshold of 10 g/dL at any given time, findings noted by others [6, 7, 11, 12] that are likely due to changes in patterns of anemia-related medication use. Our study cannot determine whether this represents an improved treatment approach compared with approaches used before the PPS; indeed, results from clinical trials indicate that lower hemoglobin levels are associated with lower risk of cardiovascular events [13, 14].

Collectively, these findings suggest that an ESA-sparing anemia-management strategy resulting in lower mean hemoglobin levels might be the best overall approach for dialysis patients, even if it results in a modest increase in transfusion rates.

The present findings should be considered in the context of transfusion trends occurring in the general population. As of 2011, there was an excess of whole blood and RBC transfusions in the US of about 5.2 % [15], a trend that appears likely to continue [16]. Overall, whole blood and RBC transfusions decreased nationally by 8.2 % in 2011 compared with 2008. Given this trend, the relative increase in transfusions in dialysis patients may be somewhat greater than it initially appears. It is very unlikely, however, that changes in transfusion practices in dialysis patients could seriously tax national blood reserves.

Thus, it is appropriate for society to debate how to optimally use this resource. This is especially true when alternative treatments are available that can partially ameliorate the condition (e.g., ESAs or IV iron) [17]. Additionally, transfusions are not without risk, as they are associated with inflammatory responses (which may in turn exacerbate other conditions) [18], sensitization (which increases the difficulty of obtaining matches for organ transplant) [19], transmission of blood-borne diseases [20], and, likely, other infections [21]. When individualizing therapy, these risks of transfusions should be balanced against the risks of other therapies. IV iron, for example, constitutes an oxidative stress and may contribute to the inflammatory milieu characteristic of dialysis patients, while injudicious use of ESAs has been associated with an increased risk of cardiovascular events.

Additionally, an increase in transfusions has implications for costs and resource use, for which setting is a major determinant. Transfusions can occur in several settings, each of which represents a unique clinical environment, results in different patient experiences, entails specific costs, and uses different reimbursement mechanisms. That transfusion use increased roughly twice as much in the observation environment compared with the ED environment may more generally reflect hospitals increasing use of the observation stay mechanism [22]. Possibly, hospitals are more readily transferring patients from the ED to the observation areas, given that transfusions typically take several hours to prepare and administer and EDs typically focus on rapid patient turnaround. However, we cannot be certain that this is the case.

In the face of increasing overall use of observation stays, the non-trivial 13.9 % increase in ED transfusions over the study period invites particularly close scrutiny. The ED is inherently a suboptimal environment in which to administer transfusions because it is cost-intensive to the facility and time- and space-limited. Transfusions typically require several hours to administer, and even if administered more quickly while the patient undergoes acute hemodialysis, the time and resource investment is substantial in the hyperacute ED setting. As a particularly expensive site at which to render care, the ED may not be the most appropriate place to address what may be, at least in part, a chronic medical issue. Whether EDs are indeed being used more often for transfusions, or whether patients are more likely to receive RBC transfusions when they arrive at an ED with an acute illness with lower mean hemoglobin concentrations is uncertain, and should be investigated.

Transfusions that occur in the inpatient setting also have unique but important cost implications. Inpatient transfusion costs, which are identified under the DRG system, cannot be directly addressed by our study design. However, inpatient transfusions represent a cost currently borne by hospitals. Unless unrecognized cost efficiencies have been realized in the inpatient RBC transfusion process, hospitals may be bearing the costs of changes in outpatient management if there have been no concomitant changes to the Medicare DRG-based reimbursement system, effectively representing cost shifting from dialysis providers and the Medicare ESRD program to hospitals. However, because we cannot directly account for inpatient costs with our present study, we cannot directly demonstrate such cost shifting.

Costs putatively borne by hospitals appear to be more than offset by overall savings to Medicare. As has been demonstrated by a US Government Accountability Office report and in several recent publications, the new PPS, coupled with the 2011 ESA label revision by the US Food and Drug Administration, resulted in an approximately 25 % reduction in ESA use compared with pre-2011 levels, similar to our estimates [23, 24]. Our findings regarding the association of the PPS and ESA label change with patient hemoglobin concentrations and RBC transfusion rates are also broadly concordant with the literature, and with CMS’ own claims-monitoring data and data from the US Renal Data System, namely an increase in the rate of RBC transfusions by 25 to 40 % (despite fluctuations driven by the completeness of the available information), a decline in facility-wide hemoglobin concentrations, and an increase in the percentage of patients with hemoglobin concentrations below 10 g/dL. While prior work has provided important estimates of the payer burden associated with outpatient RBC transfusions, including costs associated with monitoring, laboratory testing, and associated complications, it has not fully considered the effects of the bundle or the ESA label change on outpatient transfusion costs on a PPPM basis. Such costs, although publicly available via Medicare facility cost reports, which include financial data related to provider costs, revenues, and operating margins, are available only in raw format with minimal levels of analytical processing [25]. While ESAs, iron, and transfusions are complementary therapies for anemia management, each has its own unique risks and benefits. A growing tolerance for lower mean hemogloblin levels in dialysis patients likely resulted in non-trivial overall savings for Medicare, when the effects of the PPS, the Quality Improvement Program withholds, and other factors are taken into account.

Of note, we made no attempt to ascertain the specific indication for transfusions. This would be a challenging exercise, since a transfusion may occur in the setting of an acute exacerbation of another disease (such as a cardiac or pulmonary disorder), which may be coded as the principal reason for seeking treatment. As such, we cannot determine precisely why a given patient receives a transfusion. This does not, however, undermine our finding that transfusions have increased in the ED, observation, and inpatient settings. Possibly, patients who present to these settings with, on average, lower hemoglobin levels than in the past receive transfusions from providers who are less tolerant of anemia than nephrologists, for whom management of substantial anemia is a routine clinical occurrence.

Our study is subject to a number of important limitations. Our data are observational, so granular patient detail is lacking. Additionally, in more recent years, transfusion rates appear to have declined from a peak after the PPS. This could be due to providers’ increasing confidence, experience, and familiarity with lower mean hemoglobin levels in dialysis patients, or to providers adapting to hospital-led initiatives to limit blood transfusions. Thus, our work may not be predictive of the future transfusion landscape for dialysis patients. Also, billing claims are an imperfect source from which to determine how medical care (including transfusions) is rendered, since they are designed to capture the payment, rather than strictly clinical, aspects of care. Even so, we followed previously published methodology where possible [5, 8]. While it is possible that additional transfusion-related adverse events may have been recorded in 2011 relative to 2010 because of the increase in the fields available for International Classification of Diseases, Ninth Revision, Clinical Modification diagnosis codes from 10 to 25, the adverse events we examined were of substantial clinical significance, and as such unlikely to occupy positions below the top 10. Additionally, as stated, we cannot directly measure inpatient transfusion costs borne by the hospital, so it is uncertain whether these costs have indeed increased; to fully understand the payer impact of a rise in inpatient transfusions, novel approaches capable of attributing inpatient hospitalizations to the need for RBC transfusions to manage anemia in chronic kidney disease are needed. Likewise, we cannot directly measure savings attributed to less use of ESAs, since ESA costs are now subsumed in the PPS. Also, as stated, the introduction of the PPS coincided with an ESA label change, and we cannot determine how much of the changes we observed were due to introduction of the PPS or to the label change or other factors. Also, as an observational study, this study cannot definitively determine causality. For example, it is uncertain whether RBC transfusions are being administered to acutely ill patients in EDs because of changes in anemia practice patterns or for other reasons. Our study was not designed to address the potential impact of these changes on outcomes such as mortality and cardiovascular events. It may well be the case that recent changes in anemia management have had a beneficial effect on morbidity and mortality in dialysis patients; this issue awaits more definitive study. We also did not undertake specific case-mix adjustment in this analysis. Review of United States Renal Data System data suggests that the distribution of causes of ESRD, the mean age at dialysis initiation, and the spectrum of comorbidity burden did not change materially over this period, making potential changes in case mix unlikely to explain our findings. Finally, our findings are limited to patients who were covered by Medicare Parts and A and B and who were dialyzing in facilities that fully opted in to the PPS at the earliest opportunity.

## Conclusions

In conclusion, the introduction of the expanded PPS appears to be associated with less ESA and IV iron use, lower hemoglobin concentrations, and greater use of RBC transfusions. Overall Medicare payments to dialysis facilities appear to have decreased modestly. However, transfusion use has increased in both the outpatient setting (including ED visits and observation stays, which are billed as outpatient visits when patients are not subsequently admitted) and the inpatient setting. While we cannot definitively determine whether inpatient transfusion costs borne by the hospitals have increased, it appears likely that the PPS has been associated with cost shifting from dialysis facilities to hospital-based environments. No conclusions can be drawn from this study as to whether this treatment approach has benefitted or harmed patients receiving maintenance dialysis. Necessary and important future attempts to control costs should proceed with an understanding that a reduction in potentially avoidable interventions and a shift toward use of less-costly and less-acute health care settings are also important goals.

## Abbreviations

CHF: congestive heart failure
CMS: Centers for Medicare & Medicaid Services
DRG: diagnosis-related group
EPO: epoetin alfa
ESA: erythropoiesis-stimulating agent
ESRD: end-stage renal disease
IV: intravenous
PPPM: per patient per month
PPS: Prospective Payment System
RBC: red blood cell
SAF: standard analysis file

## References

[1] Centers for Medicare & Medicaid Services. Federal Register. Medicare Program; End-Stage Renal Disease Prospective Payment System; Final Rule and Proposed Rule: CMS-42 CFR Parts 410, 413 and 414. 2010. http://www.gpo.gov/fdsys/pkg/FR-2010-08-12/pdf/2010-18466.pdf. Accessed 5 Feb 2016.

[2] Centers for Medicare & Medicaid Services. Federal Register. Medicare Program; End-Stage Renal Disease Prospective Payment System, Quality Incentive Program, and Bad Debt Reductions for All Medicare Providers. 2012. https://www.federalregister.gov/articles/2012/11/09/2012-26903/medicare-program-end-stage-renal-disease-prospective-payment-system-quality-incentive-program-and. Accessed 5 Feb 2016. 23139948

[3] US Food and Drug Administration (2011). FDA modifies dosing recommendations for Erythropoiesis-Stimulating Agents.

[4] Ibrahim HN, Ishani A, Foley RN, Guo H, Liu J, Collins AJ (2008). Temporal trends in red blood transfusion among US dialysis patients, 1992-2005. Am J Kidney Dis.

[5] Shander A, Hofmann A, Ozawa S, Theusinger OM, Gombotz H, Spahn DR (2010). Activity-based costs of blood transfusions in surgical patients at four hospitals. Transfusion.

[6] Pisoni RL, Fuller DS, Bieber BA, Gillespie BW, Robinson BM (2012). The DOPPS practice monitor for US dialysis care: trends through August 2011. Am J Kidney Dis.

[7] Hirth RA, Turenne MN, Wilk AS, Wheeler JR, Sleeman KK, Zhang W (2014). Blood transfusion practices in dialysis patients in a dynamic regulatory environment. Am J Kidney Dis.

[8] Gitlin M, Lee JA, Spiegel DM, Carson JL, Song X, Custer BS (2012). Outpatient red blood cell transfusion payments among patients on chronic dialysis. BMC Nephrol.

[9] Bureau of Labor Statistics. Consumer Price Index. CPI Tables. 2012. http://www.bls.gov/cpi/tables.htm. Accessed 5 Feb 2016.

[10] Hirth RA, Turenne MN, Wheeler JR, Nahra TA, Sleeman KK, Zhang W (2013). The initial impact of Medicare’s new prospective payment system for kidney dialysis. Am J Kidney Dis.

[11] US Renal Data System: USRDS 2013 Annual Data Report: Atlas of Chronic Kidney Disease & End-Stage Renal Disease in the United States. Vol 2, Chapter 10. 2013 edition Bethesda, MD: National Institutes of Health, National Institute of Diabetes and Digestive and Kidney Diseases; 2013.

[12] Brunelli SM, Monda KL, Burkart JM, Gitlin M, Neumann PJ, Park GS (2013). Early trends from the Study to Evaluate the Prospective Payment System Impact on Small Dialysis Organizations (STEPPS). Am J Kidney Dis.

[13] Singh AK, Szczech L, Tang KL, Barnhart H, Sapp S, Wolfson M (2006). Correction of anemia with epoetin alfa in chronic kidney disease. N Engl J Med.

[14] Drueke TB, Locatelli F, Clyne N, Eckardt KU, Macdougall IC, Tsakiris D (2006). Normalization of hemoglobin level in patients with chronic kidney disease and anemia. N Engl J Med.

[15] United States Department of Health and Human Services. National Blood Collection and Utilization Survey Report, 2011. http://www.hhs.gov/ash/bloodsafety/2011-nbcus.pdf. Accessed 5 Feb 2016.

[16] Williamson LM, Devine DV (2013). Challenges in the management of the blood supply. Lancet.

[17] KDIGO (2012). KDIGO Clinical practice guideline for anemia in chronic kidney disease. Kidney Int Suppl.

[18] Neal MD, Raval JS, Triulzi DJ, Simmons RL (2013). Innate immune activation after transfusion of stored red blood cells. Transfus Med Rev.

[19] Obrador GT, Macdougall IC (2013). Effect of red cell transfusions on future kidney transplantation. Clin J Am Soc Nephrol.

[20] Turner ML, Ludlam CA (2009). An update on the assessment and management of the risk of transmission of variant Creutzfeldt-Jakob disease by blood and plasma products. Br J Haematol.

[21] Rohde JM, Dimcheff DE, Blumberg N, Saint S, Langa KM, Kuhn L (2014). Health care-associated infection after red blood cell transfusion: a systematic review and meta-analysis. JAMA.

[22] Feng Z, Wright B, Mor V (2012). Sharp rise in Medicare enrollees being held in hospitals for observation raises concerns about causes and consequences. Health Aff (Millwood).

[23] US Government Accountability Office. End-Stage Renal Disease: Reduction in Drug Utilization Suggests Bundled Payment Is Too High. Report GAO-13-190R, 2012. December 7, 2012. Washington, DC. http://www.gao.gov/assets/660/650667.pdf. Accessed 5 Feb 2016.

[24] Collins AJ, Foley RN, Herzog C, Chavers B, Gilbertson D, Herzog C et al. US Renal Data System 2012 Annual Data Report. Am J Kidney Dis. 2013;61 Suppl 1: A7, e1-A7, 476. 10.1053/j.ajkd.2012.11.031 23253259

[25] Centers for Medicaid & Medicare. Cost Reports. 2011-2013. http://www.cms.gov/Research-Statistics-Data-and-Systems/Files-for-Order/CostReports/Downloads/RNL11/RNL11-All-Years.ZIP. Accessed 5 Feb 2016.

